# An assessment of attitudes toward gender inequitable sexual and reproductive health norms in South Sudan: a community-based participatory research approach

**DOI:** 10.1186/1752-1505-7-24

**Published:** 2013-11-09

**Authors:** Jennifer Scott, Sarah Averbach, Anna Merport Modest, Michele Hacker, Sarah Cornish, Danielle Spencer, Maureen Murphy, Parveen Parmar

**Affiliations:** 1 Harvard Humanitarian Initiative, Cambridge, MA, USA; 2 Department of Medicine, Division of Women’s Health, Brigham and Women’s Hospital, Boston, MA, USA; 3 Department of Obstetrics and Gynecology, Beth Israel Deaconess Medical Center, Boston, MA, USA; 4 Department of Emergency Medicine, Division of International Health and Humanitarian Programs, Brigham and Women’s Hospital, Boston, MA, USA; 5 Department of Epidemiology, Harvard School of Public Health, Boston, MA, USA; 6 American Refugee Committee, Juba, South Sudan

**Keywords:** South Sudan, Conflict, Sexual health, Reproductive health, Gender, Inequitable norms, Gender Inequitable Men scale

## Abstract

**Background:**

Communities in South Sudan have endured decades of conflict. Protracted conflict exacerbated reproductive health disparities and gender inequities. This study, conducted prior to the country’s 2011 independence, aimed to assess attitudes toward gender inequitable norms related to sexual relationships and reproductive health and the effects of sex, age, and education on these attitudes.

**Methods:**

Applying a community-based participatory research approach and quota sampling, 680 adult male and female respondents were interviewed in seven sites within South Sudan in 2009–2011. The verbally administered survey assessed attitudes using the Gender Equitable Men scale. Data were stratified by sex, age (≤35 years and >35 years), and education.

**Results:**

Of 680 respondents, 352 were female, 326 were male, and 2 did not indicate their sex. The majority of women (77%) and men (74%) agreed “a man needs other women, even if things with his wife are fine”. Respondents who reported no education (60%) were more likely than those who reported any education (45%) to agree “if a woman is married, she should have sex with her husband whenever he wants to, even if she doesn’t want to” (p = 0.002). The majority of women (74%) and men (73%) agreed “it is a woman’s responsibility to avoid getting pregnant”. Respondents who reported no education (81%) were more likely than those who reported any education (72%) to agree with this statement (p = 0.04). When asked about condom use, the majority of respondents, across both sexes and both age groups, agreed “it would be outrageous for a wife to ask her husband to use a condom” and “women who carry condoms are easy”. There were no statistically significant differences between the two age groups for any of the assessed gender inequitable norms.

**Conclusion:**

The study reveals differences in attitudes toward gender inequitable sexual and reproductive health norms among those surveyed in South Sudan when stratified by sex and education. As a new nation seeks to strengthen its health system, these data can inform sexual and reproductive health policies and programming in South Sudan.

## Background

Communities in the newly independent Republic of South Sudan have experienced decades of conflict and displacement. Protracted conflict exacerbated health disparities and gender inequities [1,2]. Women have endured physical and sexual violence and suffered from limited health and social services [3-5]. Women and girls also face challenges of early marriage, high maternal morbidity and mortality, limited access to reproductive health services and family planning, and limited educational attainment [1-3,6,7]. Promotion of safe sexual practices and equitable reproductive health norms has the potential to improve the health of women and men in South Sudan. Data regarding attitudes toward sexual and reproductive health norms in South Sudanese communities can be used to design effective programs.

Reproductive health and rights, in addition to gender equality and women’s empowerment, are important pillars of population and development programming [8]. Populations affected by conflict and displacement often have poor access to reproductive health care and guidelines exist to ensure adequate provision of reproductive health in these contexts [9,10]. Sudan was one of 179 countries to sign the International Conference on Population and Development Programme of Action in 1994 to promote reproductive rights, including the right to make decisions concerning reproduction free of coercion, discrimination, and violence [8,11]. Equality between women and men in matters of sexual relations and reproduction, mutual respect, and shared responsibility are also emphasized in the Programme of Action [8].

Reproductive health indicators illustrate the challenges facing women in South Sudan. The 2006 Southern Sudan Household Health Survey revealed a maternal mortality ratio of 2054 per 100,000 live births [3]. According to recent estimates, the maternal mortality ratio in South Sudan continues to be one of the highest in the world [12]. There is an unmet need for contraception with a low contraceptive prevalence of 4% for any method among women aged 15–49 [13]. Among those aged 15–24, Human Immunodeficiency Virus (HIV)/Acquired Immune Deficiency Syndrome (AIDS) prevalence was more than twice as high for females (1.3%) compared to males (0.5%) [7]. The A 2010 national health survey in South Sudan found that among women aged 15–49 years, less than 9% had comprehensive knowledge of HIV, meaning they could identify at least two ways of avoiding HIV infection and reject three common misconceptions about HIV transmission [3,14]. South Sudanese medical experts note that HIV is the single disease that could compromise the future of the new nation and also highlight contributing factors to HIV transmission including high rates of illiteracy, few awareness campaigns, low condom use among young women, and the cultural acceptance of multiple sexual relationships even within marriage [14].

Following South Sudan’s independence from North Sudan in July 2011, there is greater emphasis on rebuilding the health care system and improving its health programming. The Government’s proposed strategy, known as the Health Sector Development Plan, aims to improve basic health services with a specific focus on maternal, reproductive, and child health [15,16]. In a country where the health care system has been strained by decades of conflict, over 80% of medical care is provided by non-governmental organizations [14,15].

Despite greater emphasis on strengthening sexual and reproductive health programming in South Sudan, there are limited data about the attitudes of South Sudanese women and men regarding sexual relationships and reproductive health norms. The objectives of the study were to provide data on attitudes toward gender inequitable norms related to sexual relationships and reproductive health in South Sudan and to assess whether attitudes toward these norms differed according to sex, age, or education.

## Methods

The assessment of attitudes toward sexual relationships and reproductive health was part of a larger assessment in South Sudan [17]. Assessments were conducted in seven South Sudanese sites in 2009–2011, pre-independence, as part of programming for American Refugee Committee, a non-governmental organization in South Sudan. The assessment sites included: Aweil in Northern Bahr El Ghazal State; Wau in Western Bahr El Ghazal State; Lainya, Morobo, and Ronyi in Central Equatoria State; and Malakal in Upper Nile State.

The methodology was previously described and is summarized here [17]. As community-based participatory research, a community steering committee of community members and potential program recipients participated in the study design, survey development, and data collection [18]. Each assessment site was divided into zones of approximately equal population sizes and quota sampling was applied to attain equal sex and age distribution. We aimed to sample 100 women and men in each site. Households, defined as a group of people who usually live and eat together, were randomly selected within the zone. Trained male and female South Sudanese interviewers verbally administered the survey to selected respondents. Verbal consent was obtained for adult respondents and verbal assent was obtained for respondents ≤ 18 years of age. Respondents were informed that their participation was voluntary.

The 52-question survey assessed violence, sexual relationships, reproductive health, gender norms, gender inequitable practices, and the empowerment of women. The survey included 24 questions from the Gender Equitable Men scale [19,20] to assess attitudes toward gender inequitable norms. The remaining questions were determined in collaboration with the community steering committee. Respondents were asked to “agree”, “partially agree”, or “disagree” with statements about gender norms.

Ethical approval was granted by Harvard School of Public Health. Permission to conduct the study as part of American Refugee Committee’s programming was granted by the Republic of South Sudan and the village elder in each assessment site. Interviews were conducted in a private setting in the household. Data were anonymous and stored securely. Services and referrals for respondents were provided if needed or requested.

### Statistical analysis

Statistical analysis was performed using SAS® 9.3 statistical software (SAS Institute Inc., Cary, NC). Data are reported as median and interquartile range (IQR) or proportion. We compared responses of women and men, as well as the responses across age strata (≤35 years and >35 years) and education strata (“no education” and “any education”) with the chi-squared, Fisher’s exact or Wilcoxon rank sum test. For the analysis, the responses “agree” and “partially agree” were combined. All tests were two-sided and p-values <0.05 were considered statistically significant.

## Results

A total of 697 respondents were interviewed; 17 respondents <18 years of age were excluded. The response rate for Wau was 96% (102/106). Response rates were not recorded in the other sites; however, study leaders for those sites reported few refusals and few households without an available respondent.

### Demographics

Of the 680 respondents, 52% were women, 48% were men, and 2 individuals did not indicate their sex. The majority of respondents reported to be married, Christian, and represented the major South Sudanese ethnic groups. The median age was 34.0 years (25.0-45.0). Respondents reported a median of 5.0 years (0.0-9.0) of formal (school-based) education, with men reporting more years of education than women (p < 0.0001) and a higher reading level (p < 0.0001). Respondents reporting no education were older (38.0 years) than those reporting any education (30.0 years), (p < 0.0001). Demographic characteristics are reported in Table 1.

**Table 1:** Respondent characteristics

**Characteristic**	**All respondents**	Male*	Female*	P**	**No education†**	**Any education†**	**P‡**
	**n = 680**	**n = 326**	**n = 352**		**n = 159**	**n = 444**	
**Age (years)**	34.0 (25.0-45.0)	35.0 (25.0-45.0)	33.0 (25.0-44.5)	0.21	38.0 (29.0-52.0)	30.0 (23.0-42.0)	<0.0001
**Marital Status**				<0.0001			<0.0001
**Single**	120 (17.7)	84 (25.8)	36 (10.2)		10 (6.3)	104 (23.4)	
**Married**	476 (70.0)	210 (64.4)	264 (75.0)		124 (78.0)	301 (67.8)	
**Widowed or divorced**	39 (5.7)	7 (2.2)	32 (9.1)		14 (8.8)	16 (3.6)	
**Unknown**	45 (6.6)	25 (7.7)	20 (5.7)		11 (6.9)	23 (5.2)	
**Literacy**				<0.0001			<0.0001
**Reads easily**	228 (33.5)	141 (43.2)	87 (24.7)		19 (11.9)	198 (44.6)	
**Reads with difficulty**	161 (23.7)	75 (23.0)	86 (24.3)		4 (2.5)	154 (34.7)	
**Not at all**	222 (32.6)	80 (24.5)	140 (39.8)		123 (77.4)	67 (15.1)	
**Unknown**	69 (10.1)	30 (9.2)	39 (11.1)		13 (8.2)	25 (5.6)	
**Education (years)**	5.0 (0.0-9.0)	7.0 (2.0-10.0)	4.0 (0.0-8.0)	<0.0001	0.0 (0.0-0.0)	7.0 (4.0-10.0)	<0.0001

Data are presented as n (%) or median (interquartile range).

*2 participants were missing sex data.

**Comparison is between males and females.

†77 people missing years of education.

‡Comparison is between no education and education.

### Sexual relationships

Regarding sexual relationships, the majority of both women and men agreed with the statements “it is the man who decides what type of sex to have”, “men are always ready to have sex”, and “men need sex more than women do”. (Table 2) Compared to men, women were more likely to agree with each of these statements (all p≤0.007). Women (49%) were also more likely than men (38%) to agree “you don’t talk about sex, you just do it” (p = 0.003). Among all assessed gender inequitable norms, there were no statistically significant differences between those aged ≤35 years and >35 years.

**Table 2:** Attitudes toward gender inequitable norms related to sexual relationships stratified by sex and age

**Survey question**		**Male**	**Female**	**P**	**Age ≤ 35 yrs**	**Age > 35 yrs**	**P**
		**n = 326**	**n = 352**		**n = 367**	**n = 313**	
***It is the man who decides what type of sex to have***	**Agree**	226 (69.3)	271 (77.0)	0.007	262 (71.4)	236 (75.4)	0.09
	**Disagree**	97 (29.8)	72 (20.5)		102 (27.8)	68 (21.7)	
	**Missing**	3 (0.9)	9 (2.6)		3 (0.8)	9 (2.9)	
***Men are always ready to have sex***	**Agree**	201 (61.7)	259 (73.6)	0.0007	248 (67.6)	214 (68.4)	0.68
	**Disagree**	123 (37.7)	90 (25.6)		118 (32.2)	95 (30.4)	
	**Missing**	2 (0.6)	3 (0.9)		1 (0.3)	4 (1.3)	
***Men need sex more than women do***	**Agree**	218 (66.9)	285 (81.0)	<0.0001	269 (73.3)	235 (75.1)	0.47
	**Disagree**	103 (31.6)	64 (18.2)		95 (25.9)	73 (23.3)	
	**Missing**	5 (1.5)	3 (0.9)		3 (0.8)	5 (1.6)	
***You don’t talk about sex, you just do it***	**Agree**	124 (38.0)	173 (49.1)	0.003	150 (40.9)	149 (47.6)	0.09
	**Disagree**	199 (61.0)	173 (49.1)		211 (57.5)	161 (51.4)	
	**Missing**	3 (0.9)	6 (1.7)		6 (1.6)	3 (1.0)	
***A man needs other women, even if things with his wife are fine***	**Agree**	241 (73.9)	272 (77.3)	0.25	272 (74.1)	243 (77.6)	0.26
	**Disagree**	83 (25.5)	76 (21.6)		92 (25.1)	67 (21.4)	
	**Missing**	2 (0.6)	4 (1.1)		3 (0.8)	3 (1.0)	
***If a woman is married, she should have sex with her husband whenever he wants to, even if she doesn’t want to***	**Agree**	148 (45.4)	179 (50.9)	0.17	184 (50.1)	145 (46.3)	0.35
	**Disagree**	174 (53.4)	170 (48.3)		180 (49.1)	164 (52.4)	
	**Missing**	4 (1.2)	3 (0.9)		3 (0.8)	4 (1.3)	

Data are presented as n (%).

*P-values do not include missing category.

In regard to sexual relationships within marriage, 77% of women and 74% of men agreed “a man needs other women, even if things with his wife are fine”. Furthermore, 51% of women and 45% of men surveyed agreed that “if a woman is married, she should have sex with her husband whenever he wants to, even if she doesn’t want to”. Table 3 shows that respondents who reported no education were more likely than those who reported any education to agree with these two statements (p = 0.004 and p = 0.002, respectively).

**Table 3:** Attitudes toward gender inequitable norms related to sexual relationships stratified by education

**Survey question**		**No education n = 159**	**Any education n = 444**	**P**
***It is the man who decides what type of sex to have***	**Agree**	123 (77.4)	323 (72.8)	0.31
	**Disagree**	35 (22.0)	115 (25.9)	
	**Missing**	1 (0.6)	6 (1.4)	
***Men are always ready to have sex***	**Agree**	114 (71.7)	299 (67.3)	0.29
	**Disagree**	44 (27.7)	143 (21.2)	
	**Missing**	1 (0.6)	2 (0.5)	
***Men need sex more than women do***	**Agree**	130 (81.8)	317 (71.4)	0.01
	**Disagree**	28 (17.6)	123 (27.7)	
	**Missing**	1 (0.6)	4 (0.9)	
***You don’t talk about sex, you just do it***	**Agree**	81 (50.9)	194 (43.7)	0.15
	**Disagree**	78 (49.1)	244 (55.0)	
	**Missing**	0 (0.0)	6 (1.4)	
***A man needs other women, even if things with his wife are fine***	**Agree**	134 (84.3)	321 (72.3)	0.004
	**Disagree**	25 (15.7)	120 (27.0)	
	**Missing**	0 (0.0)	3 (0.7)	
***If a woman is married, she should have sex with her husband whenever he wants to, even if she doesn’t want to***	**Agree**	95 (59.8)	201 (45.3)	0.002
	**Disagree**	63 (39.6)	239 (53.8)	
	**Missing**	1 (0.6)	4 (0.9)	

Data are presented as n (%).

*P-values do not include missing category.

### Reproductive health

The majority of respondents, 74% of women and 73% of men, agreed “it is a woman’s responsibility to avoid getting pregnant”. (Table 4) Respondents who reported no education (81%) were more likely than those who reported any education (72%) to agree with the former statement (p = 0.04), but no differences were observed when stratified by sex and age. (Tables 4 and 5) When asked about condom use, the majority of respondents, across both sexes and both age groups, agreed that “it would be outrageous for a wife to ask her husband to use a condom” and that “women who carry condoms are easy”. There were no differences in agreement for these statements when stratified by sex, age, or education (all p≥0.10).

**Table 4:** Attitudes toward gender inequitable norms related to reproductive health stratified by sex and age

**Survey question**		**Male**	**Female**	**P**	**Age ≤ 35 yrs**	**Age > 35 yrs**	**P**
		**n = 356**	**n = 352**		**n = 367**	**n = 313**	
***It is a woman’s responsibility to avoid getting pregnant***	**Agree**	239 (73.3)	261 (74.1)	0.53	263 (71.7)	239 (76.4)	0.12
	**Disagree**	27 (26.7)	85 (24.1)		102 (27.8)	70 (22.4)	
	**Missing**	0 (0.0)	6 (1.7)		2 (0.5)	4 (1.3)	
***It would be outrageous for a wife to ask her husband to use a condom***	**Agree**	185 (56.7)	219 (62.2)	0.10	222 (60.5)	184 (58.8)	0.99
	**Disagree**	136 (41.7)	124 (35.2)		142 (38.7)	118 (37.7)	
	**Missing**	5 (1.5)	9 (2.6)		3 (0.8)	11 (3.5)	
***Women who carry condoms on them are ‘easy’***	**Agree**	218 (66.9)	231 (65.6)	0.73	250 (68.1)	200 (63.9)	0.56
	**Disagree**	97 (29.8)	109 (31.0)		110 (30.0)	97 (31.0)	
	**Missing**	11 (3.4)	12 (3.4)		7 (1.9)	16 (5.1)	

Data are presented as n (%).

*P-values do not include missing category.

**Table 5:** Attitudes toward gender inequitable norms related to reproductive health stratified by education

**Survey question**		**No education**	**Any education**	**P**
		**n = 159**	**n = 444**	
***It is a woman’s responsibility to avoid getting pregnant***	**Agree**	128 (80.5)	319 (71.9)	0.04
	**Disagree**	31 (19.5)	122 (27.5)	
	**Missing**	0 (0.0)	3 (0.7)	
***It would be outrageous for a wife to ask her husband to use a condom***	**Agree**	95 (59.8)	263 (59.2)	0.99
	**Disagree**	63 (39.6)	174 (39.2)	
	**Missing**	1 (0.6)	7 (1.6)	
***Women who carry condoms on them are ‘easy’***	**Agree**	103 (64.8)	304 (68.5)	0.29
	**Disagree**	54 (34.0)	129 (29.1)	
	**Missing**	2 (1.3)	11 (2.5)	

Data are presented as n (%).

*P-values do not include missing category.

## Discussion

Our study provides data on the attitudes of both women and men in South Sudan toward gender inequitable norms related to sexual relationships and reproductive health at a time when the newly independent nation is strengthening reproductive health programs and policies. To our knowledge, there are no other published data of this nature from this time period for South Sudan, and these data contribute to a growing research field examining the role of gender equity in sexual and reproductive health.

The majority of surveyed women and men agreed with gender inequitable norms related to sexual relationships and reproductive health. Such gender inequitable norms could be a barrier to the promotion of safe sexual practices and reproductive health in South Sudan, including utilization of contraception to prevent pregnancy and sexually transmitted infections. Further studies are needed in South Sudan to understand how gender norms shape actual sexual practices and reproductive health outcomes.

Our data highlight the need for equitable dialogue and decision-making around sexual relationships in South Sudan. Among assessed attitudes toward sexual relationships, women were more likely than men to agree that men decide what type of sex to have, men are always ready to have sex, men need sex more than women do, and you don’t talk about sex. It is unclear why women would be more likely to agree with these gender inequitable norms, but it is likely a reflection of socially acceptable norms within the assessed communities.

Other studies examining the complexities of gender equity and power and health also found that in many circumstances women had more gender inequitable views than men toward certain practices [21,22]. Women’s sexual empowerment has been associated with greater utilization of family planning methods [23,24] and gender equitable attitudes have been correlated with higher modern contraceptive use [25]. There is a paucity of data for South Sudan for comparison. As part of reproductive health program implementation in South Sudan, it will be important to continue to evaluate and address differences among women and men regarding sexual relationships and to promote equitable attitudes and dialogue.

While the majority of women and men in our study agreed with gender inequitable statements regarding sexual relationships, the majority of women and men agreed it is a woman’s responsibility to avoid getting pregnant. These data suggest that men hold decision-making power around the nature of sexual relationships, but that the burden of child spacing and family planning rests on women. Such inequities in norms and expectations related to family planning would be important to address as part of reproductive health programming in South Sudan. Our analysis also showed that respondents reporting no formal education were more likely than those with any education to agree that it is a woman’s responsibility to avoid getting pregnant; suggesting that general education of both women and men may promote more equitable sexual and reproductive health attitudes and behaviors. As described in other studies [26,27], education is associated with equitable and safe sexual behaviors. Reproductive health programming in South Sudan could build upon synergies across education and health sectors.

While our study did not analyze contraceptive use in general for family planning, there were gender inequities in norms regarding condom use. These findings could support the use of female-centered reproductive health programming, such as female-controlled methods of contraception, to allow for efficacious family planning in the setting of gender inequitable norms. On the other hand, these findings could also support programming that addresses underlying gender inequity around the responsibility of family planning, encourages male involvement in family planning decision-making, and strengthens mutual communication [28,29]. Other studies have shown that husbands and male partners play an important role in contraceptive decision making [25,30]. In all likelihood, a comprehensive reproductive health program in South Sudan would require an integrated approach that empowers women, engages men, and addresses underlying gender inequitable norms regarding sexual relationships and family planning.

Gender inequitable norms have important implications for family planning and also prevention of sexually transmitted infections. Among both surveyed women and men, there was an acceptance of sex outside of marriage for men, and the majority of women and men agreed that a man needs other women, even if things with his wife are fine. While our study did not assess polygamy, a 2006 South Sudanese study revealed that 28% of South Sudanese women were in polygamous unions [3]. Whether as part of polygamous unions or sexual relationships in general, this acceptance of multiple sexual partners for men has important implications for sexually transmitted infections and health. The only method of contraception known to protect against HIV transmission is condoms and experts indicate that HIV is an emerging threat in South Sudan [14]. The majority of women and men in this study, however, agreed that it would be outrageous for a wife to ask her husband to use a condom and that women who carry condoms are considered easy. Our findings raise the concern that a woman may not have power in a relationship to negotiate condom use with her partner and that programming addressing gender inequity in contraceptive decision making could help increase contraceptive uptake and empower women to utilize methods such as condoms to prevent HIV acquisition.

In recent years, there has been greater emphasis on integrating a gender equitable approach into reproductive health and HIV/AIDS programming to improve the quality and utilization of services, the sustainability of programs, and to enhance synergies across development strategies and sectors [31]. Gender equity has been shown to positively impact reproductive health outcomes such as improved contraceptive prevalence and reduced total fertility, decreased HIV transmission, and decreased maternal morbidity and mortality [31]. Aligned with the recommendation for integrating gender into programming, our data highlight the differences between women and men toward gender inequitable norms related to sexual and reproductive health that may help to inform future program planning, implementation, and evaluation in South Sudan.

The emphasis on integrating gender into reproductive health programming challenges us as researchers to consider how to best assess differences and inequities between women and men in regard to sexual and reproductive health norms. In our study, we assessed differences in attitudes between women and men using previously constructed inequitable statements as part of the Gender Equitable Men scale. However, these Gender Equitable Men scale statements do not assess how a “gender equitable woman” would approach sexual relationships and reproductive health, nor do these statements encompass all areas of sexual relationships and practices or assess reproductive health outcomes. Other scales have been used to understand gender equitable attitudes in relationship to sexual and reproductive health, such as the Household Decision-Making scale, Attitudes Toward Wife Refusing Sex scale [22], the Sexual and Reproductive Power scale [30], or unique questions to assess gender dynamics [21]. Further research and consensus on how to most accurately assess gender inequity in regard to sexual and reproductive health and how to promote gender equity in these areas are needed.

## Limitations

While our sampling strategy was designed to obtain a representative sample, quota sampling reflects the attitudes of the surveyed communities of South Sudan and may not be generalizable. Furthermore, we could not calculate response rates for most sites, and even though the interviewers reported that there were few skipped households and few who declined to be interviewed, it is possible that those who were interviewed differed from those who did not respond or who were not available. Given that an interviewer administered the survey, the interviewer’s sex or age may have influenced responses. For instance, social desirability bias may have been introduced because women and men would be more likely to report socially desirable attitudes to an interviewer of the same sex. In addition, the questions addressed sensitive issues and respondents may have exaggerated or underreported their attitudes. The political climate of South Sudan prior to independence also could have influenced responses. Our study assessed attitudes toward gender inequitable norms related to sexual relationships and reproductive health; however, our study did not assess sexual practices and reproductive health outcomes and thus, we cannot make conclusions about how differences in attitudes may impact actual behaviors and practices.

## Conclusion

Following independence in July 2011, South Sudan has opportunities for promoting gender equity and sexual and reproductive health. Sexual and reproductive health programming and policies in South Sudan should consider underlying gender dynamics, the attitudes of both women and men, and continue to promote gender equitable norms regarding sexual relationships and shared reproductive health decision making.

## Abbreviations

AIDS: Acquired Immune Deficiency Syndrome; HIV: Human Immunodeficiency Virus.

## Competing interests

The co-authors declare no competing interests.

## Authors’ contributions

JS contributed to study design, data collection, and data entry; directed data analysis; interpreted data; and wrote the initial manuscript draft. SA assisted in data interpretation and manuscript writing. AMM conducted the data analysis and contributed to data interpretation and manuscript writing. MH assisted in study design, supervised data analysis, and contributed to data interpretation and manuscript writing. SC and DS contributed to study design, data collection and interpretation, and manuscript writing. MM provided project support and contributed to data interpretation and manuscript writing. PP assisted with data interpretation and manuscript writing. All authors read and approved the final manuscript.
